# Evaluation of the clinical efficacy of microwave ablation for benign thyroid nodules based on contrast-enhanced ultrasound

**DOI:** 10.1097/MD.0000000000040774

**Published:** 2024-12-06

**Authors:** Yun Zhang, Jun Feng, Gang Fu

**Affiliations:** a Department of Ultrasonography, Jiangnan University Medical Center (Wuxi Second People’s Hospital), Jiangsu, China.

**Keywords:** benign thyroid nodules, clinical efficacy, contrast-enhanced ultrasound, microwave ablation

## Abstract

This study evaluates the clinical efficacy of microwave ablation in patients with benign thyroid nodules based on contrast-enhanced ultrasound. A total of 92 patients with benign thyroid nodules admitted to our hospital from January 2020 to December 2022 were selected as research subjects and divided into control group and observation group according to different treatment methods, with 46 cases in each group. All patients received microwave ablation. Imaging examination and monitoring were performed before and after microwave ablation. The control group underwent routine ultrasound examination, and the observation group underwent contrast-enhanced ultrasound examination. It was determined whether or not to terminate ablation therapy according to the imaging examination results. Results of enhanced MRI were used as the gold standard to evaluate the clinical efficacy, thyroid nodule volume, thyroid nodule volume reduction rate, nodule recurrence, and complication rate of the 2 groups. Using the results of enhanced MRI as the gold standard, the total ablation rate of observation group was 96.55%, which was significantly higher than that of control group 85.96%, and the nodule survival rate of 3.45% was significantly lower than that of control group 14.04% (*P* < .05). After 1 month, 3 months, 6 months, and 12 months, the thyroid nodule volume of both groups was significantly reduced, and the thyroid nodule volume of observation group was significantly smaller than that of control group (*P* < .05). After 1 month, 3 months, 6 months, and 12 months, the reduction rate of thyroid nodule volume in 2 groups was significantly increased, and the reduction rate of thyroid nodule volume in observation group was significantly higher than that in control group (*P* < .05). After treatment, the recurrence rate of nodule in observation group was 4.35%, which was significantly lower than that in control group, 15.22% (*P* < .05). After treatment, the complication rate of observation group was 8.70%, which was significantly lower than that of control group 26.09% (*P* < .05). Contrast-enhanced ultrasound can effectively monitor the treatment range of benign thyroid nodules by microwave ablation, improve clinical efficacy, reduce the recurrence rate of nodules, and has high effectiveness and safety.

## 1. Introduction

As an important endocrine organ of the body, the thyroid is involved in the metabolic process and various physiological activities through the synthesis and secretion of thyroid hormone. With the changes in lifestyle and the development of imaging technology, the detection rate of thyroid disease is gradually increasing. Among them, thyroid nodules, as a common thyroid disease, can occur in people of all ages, and the incidence rate of female is significantly higher than that of male. At present, the incidence rate of this disease has reached 20% and has been increasing year by year.^[1,2]^ Thyroid nodules are endocrine system diseases clinically characterized by localized masses in normal thyroid tissues. The etiology and pathogenesis remain unclear, which may be related to iodine intake and absorption, long-term smoking, obesity, and mental stress.^[3]^ In the early stage of the disease, there is no significant symptom. It is often found during accidental physical examination that clinical symptoms to different degrees will appear when the nodules grow larger, compress or invade the peripheral nerve tissue, including cough, hemoptysis, dysphonia, and dysphagia.^[4,5]^ Clinical examination^[6]^ has shown that most thyroid nodules are benign, and only 3% to 5% of nodules can be converted into malignant nodules. Therefore, early treatment is of great significance to improve the prognosis of patients with benign thyroid nodules.

Surgical resection, as a common treatment for benign thyroid nodules, can effectively remove the lesion tissue and alleviate the nodule compression symptoms. However, due to the large wound surface, obvious scars are easily left, which affects the aesthetic appearance. Moreover, postoperative complications are easy to occur, including hypothyroidism and recurrent laryngeal nerve injury, thus reducing the prognosis.^[7]^ With the clinical application of minimally invasive surgery, microwave ablation, as a new type of minimally invasive surgery, can observe the size, morphology, margin, internal echo, and blood flow of the lesion tissue under the guidance of ultrasound, which is conducive to the complete ablation of the lesion tissue. It has the advantages of small wound surface, high efficiency, and rapid recovery after surgery. In addition, it has a high therapeutic effect, which can maximize the protection of thyroid function.^[8]^ At present, microwave ablation is still in the early stage of exploration, and there is no accurate data to support the indications, contraindications, clinical efficacy, and postoperative complications of microwave ablation. Conventional ultrasound, color Doppler ultrasound, and other imaging techniques are mostly used to evaluate the clinical efficacy of microwave ablation in clinic, with a high recurrence rate after surgery.^[9,10]^ Contrast-enhanced ultrasound (CEUS) is a cutting-edge imaging technology that can reflect the blood perfusion and microcirculation inside the lesion tissue, and has played an important role in the diagnosis of benign and malignant thyroid diseases. However, its application to microwave ablation is still controversial.^[11]^ In view of the above situation, in this study, 92 patients with thyroid benign nodules admitted to our hospital from January 2020 to December 2022 were selected, and routine ultrasound and contrast-enhanced ultrasound monitoring were used to guide and evaluate the clinical efficacy during microwave ablation, aiming to explore the application value of contrast-enhanced ultrasound technology in the treatment of thyroid benign nodules. It is now reported as follows.

## 2. Materials and methods

### 2.1. General material

The study was approved by the Ethics Committee of Wuxi Second People’s Hospital. In this study, 92 patients with benign thyroid nodules who were admitted to our hospital from January 2020 to December 2022 were selected as the research subjects. They were divided into the control group and the observation group according to the different treatment methods, with 46 cases in each group. There was no significant difference in general information such as gender and age between the 2 groups (*P* > .05), indicating that they were comparable. All the patients were treated with microwave ablation. Imaging examination and monitoring guidance were conducted before and after microwave ablation. The control group was treated with conventional ultrasound examination, and the observation group was treated with contrast-enhanced ultrasound examination. This study was approved by the Medical Ethics Committee of our hospital.

### 2.2. Criteria of inclusion and exclusion

Inclusion criteria: ① patients who met the diagnostic criteria for thyroid benign nodules in the Expert Consensus on Thermal Ablation Treatment of Thyroid Benign Nodules, Microcarcinoma and Cervical Metastatic Lymph Nodes (2018 Version)^[12]^; ② thyroid benign nodule is confirmed through cytology and preoperative histopathological examination; ③ routine ultrasound examination and contrast-enhanced ultrasound examination of thyroid gland are performed before operation, with no contraindication for examination; ④ patients receiving microwave ablation without contraindication to surgery; ⑤ patients with normal thyroid and parathyroid function; ⑥ patients with no cognitive or mental disorders; ⑦ the patients and relatives were informed of the research content and signed the informed consent form voluntarily.

Exclusion criteria: ① patients with severe organ and tissue disorders such as cardiovascular and cerebrovascular diseases, liver and kidney function, etc; ② patients with hyperthyroidism, malignant thyroid nodules, and malignant tumors; ③ patients with past history of neck surgery; ④ patients with abnormal coagulation function, lateral recurrent laryngeal nerve function, and autoimmune system; ⑤ patients with severe infection; ⑥ patients with missing clinical data and poor compliance.

### 2.3. Research methods

Preoperative: all patients were examined by conventional two-dimensional ultrasound in the supine position, with the neck fully exposed. Linear array probe was used to scan from the horizontal line of the mandible to the suprasternal fossa, and longitudinal and transverse scans were performed to determine the size, number, location, and blood supply of thyroid nodules and their relationship to the surrounding tissues. On the basis of the above-mentioned findings, CEUS was performed in the observation group using Mindray 7S color Doppler ultrasound diagnostic apparatus (SN: 7Y-98000320), with probe models of L11-3U, and probe frequency of 9.0 MHZ, equipped with high frame rate contrast technology. After 1 sulfur hexafluoride microbubble Contrast agent (Bracco, Italy, 59 mg per bottle of SF6 gas and 25 mg of lyophilized powder) and 5 mL of normal saline were fully and uniformly shaken, 1 mL of intravenous rapid bolus injection through the elbow vein was extracted, and then 5 mL of normal saline was added. After the Contrast key and the timing key were pressed, the imaging image of the thyroid nodule was observed. The image was acquired for 5 minutes and stored, and the blood flow interest of the nodule was observed through the contrast ultrasound image.

Microwave ablation: KY-2000A microwave ablation therapeutic apparatus (Nanjing Kangyou Medical Technology Co., Ltd.) was used. The disposable ablation needle was KY-2450A-1 of Nanjing Kangyou, with the operating frequency of 2450 MHZ and microwave output power of 35 to 40 W. The patient was guided to take the supine position with a soft pillow on the shoulder, and the head was maintained in the supine position. The neck was fully exposed, and the skin in the operation area was disinfected and covered conventionally. The skin, needle tract, and the surrounding tissues of the nodule were locally anesthetized with 1% lidocaine, and the mixture of normal saline and lidocaine was injected into the periphery of the thyroid capsule, including the gap between the thyroid and the running intervals of the carotid artery, trachea, esophagus, and recurrent laryngeal nerve, to form a 0.5 to 1.0 cm liquid partition zone. An eye of a needle about 1 mm was made at the surgical site. Under the guidance of ultrasound, 15G microwave ablation needle was inserted into the thyroid nodule. According to the size and location of the nodule in the preoperative ultrasound image, mobile ablation was performed on all aspects of the thyroid nodule, and the output power was maintained at 35 to 40 W until the strong thermal energy echo completely covered or exceeded the edge of the lesion. For patients with cystic liquefaction, the fluid in the cyst should be extracted in advance to ablate the cyst wall and substantive components; if any discomfort occurs during the operation, the ablation can be stopped and the ablation protocol can be adjusted. During the operation, the observation group was used to observe the contrast-enhanced ultrasound site. The ablation method: the mobile ablation technology was used for multi-surface, multi-point, and mobile ablation in sequence from deep to shallow and from far to near, until the high-echo gasified area completely covered the nodules.

Postoperative: the control group was treated after ensuring no blood flow signal, and local pressure was applied with the dressing for 15 minutes. After the strong echo completely subsided in the observation group, contrast-enhanced ultrasound was performed again to observe the range of the filling defect area. If the contrast-enhanced ultrasound defect area ≥ ablation nodule and there was no contrast agent perfusion, the ablation was complete and the surgery was completed. If the enhanced area was still present, supplemental ablation of the enhanced area was performed until no contrast agent was infused, and the area was then pressed with the dressing for 15 minutes.

### 2.4. Observational index

① Clinical efficacy: the results of enhanced magnetic resonance imaging (MRI) were used as the gold standard to judge the complete ablation and nodule residual status of benign thyroid nodules after microwave ablation. ② Thyroid nodule volume: the thyroid nodule volumes of the 2 groups were compared before operation, and 1 month, 3 months, 6 months, and 12 months after operation. The calculation formula was as follows: nodule volume = anteroposterior diameter × left-right diameter × upper and lower diameter × π/6. ③ Thyroid nodule volume reduction rate: the thyroid nodule volume reduction rates of the 2 groups in 1 month, 3 months, 6 months, and 12 months after operation were compared, and the calculation formula was as follows: thyroid nodule volume reduction rate = (preoperative volume − volume at follow-up time)/preoperative volume × 100%. ④ Nodular recurrence: the recurrence of the nodule after 12 months of operation was counted, and the criteria for nodule recurrence^[13]^: if a patient presented a nodule with the echo similar to that of the original ablated nodule within 2 mm around the original ablated area, the nodule with the same pathological examination result and the presence of blood flow signal in the nodule, it was determined to be in situ recurrence; if there was no blood flow signal, it was considered to be a necrotic nodule that had not been absorbed after the operation, and it was determined that the nodule had not relapsed. ⑤ Complications: the complications such as nausea and vomiting, hoarse voice, neck hematoma, skin burn, and wound infection in the 2 groups were recorded.

### 2.5. Statistical methods

SPSS 24.0 statistical software was used. The data conforming to the normal distribution were measured and expressed as ($\bar{x}\pm s$). The data between groups were compared with *t* test. Enumeration data were expressed as case number (N) and percentage (%). Intergroup comparison was performed using χ^2^ test, and *P* < .05 indicated that the difference had statistical significance.

## 3. Results

### 3.1. Comparison of general material between the 2 groups

Table 1 presents a comparison of baseline characteristics between the control and observation groups, each consisting of 46 patients. The gender distribution in both groups is similar, with the control group comprising 9 males (19.57%) and 37 females (80.43%), while the observation group has 8 males (17.39%) and 38 females (82.61%), showing no statistically significant difference (*P* = .187). The average age is nearly identical, with the control group at 39.60 ± 7.65 years and the observation group at 39.64 ± 7.68 years, also lacking statistical significance (*P* = .365). Body mass index (BMI) is comparable, recorded at 23.60 ± 1.94 kg/m² in the control group and 23.67 ± 1.95 kg/m² in the observation group (*P* = .164).

**Table 1:** Comparison of general material between the 2 groups.

Group		Control group (n = 46)	Observation group (n = 46)	t value	*P* value
Gender (cases)	Male	9 (19.57)	8 (17.39)	5.306	.187
	Female	37 (80.43)	38 (82.61)		
Age (years)		39.60 ± 7.65	39.64 ± 7.68	4.689	.365
BMI (kg/m^2^)		23.60 ± 1.94	23.67 ± 1.95	7.623	.164
Lesion diameter (cm)		3.09 ± 1.06	3.10 ± 1.04	4.603	.182
Number of nodules (cases)	Single	49 (85.96)	48 (82.76)	5.490	.503
	Multiple	8 (14.04)	10 (17.24)		
Nodule location (cases)	Left	21 (36.84)	20 (34.48)	6.037	.096
	Right	34 (59.65)	36 (62.07)		
	Isthmus	2 (3.51)	2 (3.45)		
Nodule type (cases)	Mixed echo nodule	33 (57.89)	34 (58.62)	7.134	.078
	Hypoechoic nodule	24 (42.11)	24 (41.38)		

The average lesion diameter is similar between the 2 groups, with a mean of 3.09 ± 1.06 cm in the control group and 3.10 ± 1.04 cm in the observation group, and this difference is not statistically significant (*P* = .182). Regarding the number of nodules, the majority of patients in both groups present with a single nodule, comprising 85.96% in the control group and 82.76% in the observation group, while multiple nodules are present in 14.04% of control group patients and 17.24% of observation group patients, without a statistically significant difference (*P* = .503).

Nodule location is also evenly distributed between groups, with left-sided nodules in 36.84% of the control group and 34.48% of the observation group, right-sided nodules in 59.65% of the control group and 62.07% of the observation group, and isthmus nodules in 3.51% and 3.45% of patients in the control and observation groups, respectively (*P* = .096). The type of nodule is primarily mixed echo in both groups, with 57.89% in the control group and 58.62% in the observation group, while hypoechoic nodules are seen in 42.11% of the control group and 41.38% of the observation group, again showing no statistically significant difference (*P* = .078). These findings indicate that the control and observation groups are well-matched across all baseline characteristics, supporting the comparability of the 2 groups for further analysis.

### 3.2. Comparison of clinical efficacy between the 2 groups

Postoperative enhanced MRI results were taken as the gold standard. The results showed that the complete ablation rate in the observation group was 96.55%, significantly higher than 85.96% in the control group, and the nodule residual rate was 3.45%, significantly lower than 14.04% in the control group. The differences were statistically significant (*P* < .05), as shown in Table 2.

**Table 2:** Comparison of clinical efficacy between the 2 groups (cases, %).

Group	Control group (n = 46)	Observation group (n = 46)	t value	*P* value
Pathological nodule (cases)	57	58	–	–
Complete ablation nodule (cases)	49	56	–	–
Complete ablation rate	85.96%	96.55%	4.623	.010
Nodular survival rate	14.04%	3.45%	5.031	.041

*Note*: compared with the control group, ^#^*P* < .05.

### 3.3. Comparison of thyroid nodule volume between 2 groups

The results showed that there was no significant difference in thyroid nodule volume between the 2 groups before operation (*P* > .05). After 1 month, 3 months, 6 months, and 12 months, the volume of thyroid nodules in the observation group was significantly smaller than that in the control group, with statistical significance (*P* < .05), as shown in Table 3.

**Table 3:** Comparison of thyroid nodule volume between 2 groups ($\bar{x}\pm \;s$ cm^3^).

Group	Control group (n = 46)	Observation group (n = 46)	t value	*P* value
Before operation	7.69 ± 0.83	7.67 ± 0.84	3.695	.092
1 month after operation	7.35 ± 0.79	6.35 ± 0.72	5.062	.028
3 months after operation	6.38 ± 0.70	5.71 ± 0.60	5.306	.036
6 months after operation	5.62 ± 0.65	4.46 ± 0.58	2.904	.007
12 months after operation	3.68 ± 0.46	2.34 ± 0.17	5.014	.002

### 3.4. Comparison of thyroid nodule volume reduction rate between 2 groups

The results showed that after 1 month, 3 months, 6 months, and 12 months after operation, the volume reduction rate of thyroid nodules in the observation group was significantly higher than that in the control group, with statistical significance (*P* < .05), as shown in Table 4.

**Table 4:** Comparison of thyroid nodule volume reduction rate between the 2 groups ($\bar{x}\pm \;s$, %).

Group	Control group (n = 46)	Observation group (n = 46)	t value	*P* value
1 month after operation	4.46 ± 2.13	17.44 ± 4.29	6.336	.032
3 months after operation	17.45 ± 8.31	26.47 ± 11.25	5.694	.001
6 months after operation	27.08 ± 11.21	42.40 ± 12.46	7.625	.004
12 months after operation	52.47 ± 18.43	69.28 ± 37.39	5.098	.005

### 3.5. Comparison of recurrence rate of nodules between 2 groups

The results showed that the recurrence rate of nodules in the observation group was 4.35% significantly lower than that in the control group (15.22%), and the difference was statistically significant (*P* < .05), as shown in Table 5.

**Table 5:** Comparison of recurrence rate of nodules between 2 groups (cases, %).

Group	Control group (n = 46)	Observation group (n = 46)	χ^2^ value	*P* value
Recurrence of nodules	7	2	–	–
Recurrence rate of nodules	15.22%	4.35%	5.623	.006

### 3.6. Comparison of complication rate between 2 groups

Table 6 shows the difference in complication rates between the control and observation groups. In the control group, the incidence rates of nausea and vomiting, hoarse voice, neck hematoma, and wound infection were 10.87%, 4.35%, 6.52%, and 4.35%, respectively, while in the observation group, these rates were 6.52%, 0%, 2.17%, and 0%. No cases of skin burn were reported in either group. Overall, the total complication rate was 26.09% in the control group and only 8.70% in the observation group. The chi-square test result (χ² = 5.623, *P* = .034) indicates that the complication rate in the observation group was significantly lower than in the control group, suggesting a notable advantage in complication control for the observation group.

**Table 6:** Comparison of complication rate between 2 groups (cases, %).

Group	Control group (n = 46)	Observation group (n = 46)	χ^2^ value	*P* value
Nausea and vomiting	5 (10.87)	3 (6.52)	–	–
Hoarse voice	2 (4.35)	0 (0.00)	–	–
Neck hematoma	3 (6.52)	1 (2.17)	–	–
Skin burn	0 (0.00)	0 (0.00)	–	–
Wound infection	2 (4.35)	0 (0.00)	–	–
Incidence of complications	26.09%	8.70%	5.623	.034

## 4. Discussion

Benign thyroid nodules, as a common endocrine system disease, are mostly benign nodules, but a few of them may become malignant. Clinically, there is no need for too much intervention for benign thyroid nodules, and regular follow-up or review is enough. Malignant nodules are mostly treated by surgery.^[14]^ However, when the benign nodules grow to oppress the surrounding tissues or nerves, affect the appearance, and even have the possibility of malignant transformation, it is necessary to choose appropriate treatment methods, including drug treatment, surgical treatment, ablation treatment, etc, among which the drug treatment scheme is more complicated and the clinical efficacy is controversial; the traditional surgical treatment needs to make a long incision in the patient’s neck, and the thyroid tissue needs to be removed during the operation, which leads to the damage of normal thyroid function and surrounding tissue cells, and the amount of bleeding during the operation increases, and it is impossible to remove all the nodules at 1 time, which will increase the incidence of postoperative complications and affect the aesthetic degree.^[15]^ Microwave ablation, as one of the important means of minimally invasive treatment, can accurately place the microwave ablation needle into the lesion tissue under the guidance of imaging equipment, and rapidly heat to above 60 C under the action of high-frequency electromagnetic waves, resulting in coagulative necrosis of the lesion tissue, and extremely low damage to the surrounding normal tissue. With the advantages of minimal invasion, slight pain, high safety, high stability, and rapid postoperative recovery, it has been rapidly and widely used in the diagnosis and treatment of benign thyroid nodules.^[16,17]^ Ultrasound examination, as a common imaging guidance technology, can guide the ablation needle to the lesion tissue in microwave ablation, which is the key examination for the diagnosis and treatment of thyroid nodules. However, the evaluation of the ablation lesions and clinical efficacy is still in the exploratory stage.^[18]^ CEUS is to inject the contrast agent into the targeted tissue of the body by using the great difference between the acoustic characteristics of the contrast agent and the human tissue, so as to increase the ultrasonic image resolution of the targeted tissue, which has high sensitivity and specificity.^[19]^ In this study, CEUS was used in microwave ablation of thyroid benign nodules to effectively monitor the ablation range and improve the accuracy of the ablation range, thereby reducing the postoperative recurrence rate and providing guidance for the postoperative ablation effect.

Microwave ablation of thyroid nodules refers to that under the guidance of ultrasound, the ablation needle is placed into the targeted thyroid nodules, which is converted into heat energy by emitting high-frequency electromagnetic waves, leading to the coagulative necrosis of the targeted tissues, which is then absorbed by the body to disappear, thus achieving a good therapeutic effect. It has the advantages of small trauma, rapid recovery after surgery, high safety, and the like, while retaining the thyroid function to the maximum extent and maintaining the aesthetic appearance of the neck.^[20,21]^ The microwave ablation process needs to be operated under the guidance of imaging examination technology, and ultrasonic examination has the characteristics of convenient operation, safety, and real-time display. The microwave ablation based on ultrasonic examination has the following advantages: ultrasonic examination can accurately locate the position of thyroid nodules, observe the size, shape, boundary and relationship with surrounding tissues, provide image data for making the best ablation plan, improve operation accuracy and operation efficiency, reduce the risk of damage to surrounding normal tissues and improve treatment safety. In addition, ultrasound-guided targeted inactivation of nodular tissue can focus on the ablation area and scope in real time, so as to promote coagulation necrosis of targeted tissue cells and achieve the advantage of complete ablation of focal tissue with high repeatability. Through the puncture operation, the amount of bleeding during the operation is reduced, the risk of incision infection is reduced after the operation, there is no surgical scar, and the appearance is improved.^[22–24]^ Conventional ultrasound mainly relies on targeted cell echo, tumor edge, and lesion tissue ablation range to evaluate its therapeutic effect, but because the ablation process will lead to tissue gasification, it is difficult to accurately judge necrotic areas and nodules, resulting in incomplete ablation, reducing the complete ablation rate, and increasing the risk of recurrence.^[25]^ CEUS is based on the distribution of blood vessels in the focus tissue, and uses the second harmonic signal generated by the ultrasonic resonance of blood pool developer microbubbles to image, which can promote the backscattering of echo and significantly enhance the signal-to-noise ratio, clearly and sensitively observe the dynamic blood perfusion of thyroid nodules, which can be in sharp contrast with the vascular filling defect of surrounding normal tissues, and provide imaging basis for the formulation of microwave ablation scheme. It can identify the distribution of microvessels in nodules and guide microwave ablation of “portal” areas. It can monitor and guide the microwave ablation operation, evaluate the perfusion state, texture and size of blood vessels, display the distribution of blood vessels in the ablation area, judge whether the ablation is complete, end the ablation process in time, and improve the accuracy and efficiency of microwave ablation.^[26,27]^ Erturk MS et al^[28]^ take microwave ablation treatment for patients with benign thyroid nodules, which can significantly improve the nodule volume reduction rate and improve the short-term and long-term effects of disease treatment, with the advantages of less trauma and fewer complications. The results of this study show that the complete ablation rate in the observation group is significantly higher than that in the control group, and the residual rate of nodules is significantly lower than that in the control group. After 1 month, 3 months, 6 months, and 12 months, the volume of thyroid nodules in both groups was significantly reduced, and the volume of thyroid nodules in the observation group was significantly smaller than that in the control group. After 1 month, 3 months, 6 months, and 12 months, the volume reduction rate of thyroid nodules in both groups increased significantly, and the volume reduction rate of thyroid nodules in the observation group was significantly higher than that in the control group. Consistent with the above research results, contrast-enhanced ultrasound can effectively evaluate whether benign thyroid nodules are complete after microwave ablation, and improve the efficiency and accuracy of disease treatment.

Microwave ablation for benign thyroid nodules also has some defects, and postoperative complications may occur, such as nausea and vomiting, hoarseness, neck hematoma, skin burns, etc. In this study, a very small number of patients have symptoms such as nausea and vomiting, neck hematoma, etc, which may be due to the fact that the lateral part of the neck is used as the needle point for microwave ablation, and the thyroid nodules are located on the back inside. During the operation, the liquid isolation belt moves and squeezes the recurrent laryngeal nerve, resulting in postoperative complications, which are relieved by itself. In addition, after ablation, the focus tissue will recur at the edge of the original ablation area. Recurrent nodules are mostly found in nodules with rich blood supply and large volume. It may be that the heat of microwave ablation will be dispersed due to the influence of rich blood flow, which will reduce the overall ablation effect. In addition, the ablation range is small, and it is difficult for ablation heat to cover distant or biased nodule areas, which makes it difficult to completely inactivate nodules.^[29]^ For large focus tissues with rich blood flow, contrast-enhanced ultrasound can monitor the blood perfusion of thyroid nodules in real time, compare the blood perfusion of surrounding tissues in time, clearly limit the actual ablation necrosis range, find inactivated tissues in time, and carry out multiple mobile ablation and supplementary ablation to reduce the possibility of postoperative recurrence. Moreover, ultrasound contrast agent belongs to blood pool developer and will not spread to the surrounding tissues with blood flow, which improves the treatment safety and is of great value to evaluate the complete ablation of nodules and postoperative recurrence.^[30]^ The data of this study pointed out that the recurrence rate of nodules in the observation group was significantly lower than that in the control group after treatment. After treatment, the incidence of complications in observation group was significantly lower than that in control group. It shows that microwave ablation of benign thyroid nodules under contrast-enhanced ultrasound can improve the efficiency and safety of treatment and reduce the risk of recurrence of nodules after operation.

There are still some limitations in this study, mainly because the sample size is small, the sampling range is limited to our hospital, and there may be some bias between the experimental data and the actual data, which will affect the wide validity of the research results. The difference between pathological examination results and contrast-enhanced ultrasound results was not analyzed. The follow-up observation time is short, and the long-term follow-up of benign nodules has not been carried out. Therefore, the application value of contrast-enhanced ultrasound in thyroid nodules needs to be further studied.

To sum up, using contrast-enhanced ultrasound to evaluate the ablation range of benign thyroid nodules during microwave ablation treatment can effectively evaluate whether they are completely ablated, improve the complete ablation rate, reduce the recurrence rate of nodules and the incidence of complications, and provide guidance for improving the treatment effect and prognosis effect, which is worth popularizing.

## Author contributions

**Conceptualization:** Yun Zhang, Jun Feng, Gang Fu.

**Data curation:** Yun Zhang, Jun Feng, Gang Fu.

**Formal analysis:** Yun Zhang, Jun Feng, Gang Fu.

**Funding acquisition:** Gang Fu.

**Investigation:** Yun Zhang, Jun Feng, Gang Fu.

**Methodology:** Yun Zhang, Jun Feng, Gang Fu.

**Validation:** Gang Fu.

**Writing – original draft:** Yun Zhang, Gang Fu.

**Writing – review & editing:** Yun Zhang, Gang Fu.
